# Temperature along an elevation gradient determines Galapagos tortoise sex ratios

**DOI:** 10.1002/ece3.10008

**Published:** 2023-04-19

**Authors:** Sharon L. Deem, Sam Rivera, Ainoa Nieto‐Claudin, Evan Emmel, Freddy Cabrera, Stephen Blake

**Affiliations:** ^1^ One Government Drive Saint Louis Zoo Institute for Conservation Medicine St. Louis Missouri USA; ^2^ Charles Darwin Foundation Santa Cruz Galapagos Islands Ecuador; ^3^ Department of Animal Health Zoo Atlanta Atlanta Georgia USA; ^4^ The Maritime Aquarium at Norwalk Norwalk Connecticut USA; ^5^ Department of Biology Saint Louis University St. Louis Missouri USA; ^6^ Max Planck Institute of Animal Behavior Radolfzell Germany

**Keywords:** coelioscopy, conservation, iButton thermocrons, nesting zones, temperature‐dependent sex determination

## Abstract

Climate change threatens endemic island ectothermic reptiles that display small population sizes and temperature‐dependent sex determination (TSD). Studies of captive Galapagos tortoises demonstrate type A TSD with warmer incubation temperatures producing females. However, there are few published data from free‐living Galapagos tortoises on incubation temperature regimes, and none on hatchling sex ratios in the wild or the potential impacts of climate change on future sex ratios. We sought to address these deficits by quantifying incubation temperatures of nests and sex ratios of juvenile tortoises along an elevation gradient on Santa Cruz Island. We focused on three geographically separated nesting zones with mean elevations of 14 m (lower), 57 m (middle), and 107 m (upper) above sea level. Nest temperatures in 54 nests distributed across the three nesting zones were measured every 4 h throughout the incubation period using iButton thermochrons. We used coelioscopy to conduct visual exams of gonads to determine the sex of 40 juvenile tortoises from the three nesting zones. During the middle trimester of incubation, the period during which sex is determined in turtles, mean nest temperatures were 25.75°C (SD = 1.08) in the upper zone, and 27.02°C (SD = 1.09), and 27.09°C (SD = 0.85) in the middle and lower zones, respectively. The proportion of juveniles that was male increased from 11.1% in the lower zone and 9.5% in the middle zone, to 80% in the upper zone. A ca. 50 m increase in elevation induced a decrease of >1.25°C in mean nest temperature during the second trimester of incubation. Over the same elevation change, the proportion of males in the juvenile tortoise population increased by ca. 70%. Temperatures on Galapagos are predicted to increase by 1‐4°C over the next 50 years, which is likely to increase the frequency of female tortoises across the archipelago.

## Graphical abstract

We sought to improve our understanding of the impact of climate on the sex ratio of free‐living Galapagos tortoises; a species with temperature‐dependent sex determination, by quantifying sex ratios of juvenile tortoises and nest temperatures along an elevation gradient on Santa Cruz Island. A ca. 50 m increase in elevation induced a decrease in > 1.25°C in mean nest temperature in the second third of incubation. Over the same elevation change, the proportion of males in the juvenile tortoise population increased by ca. 70%.

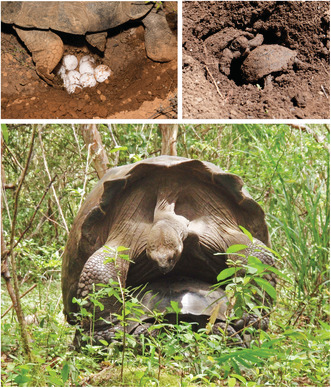

## 1 INTRODUCTION

**FIGURE 1:**
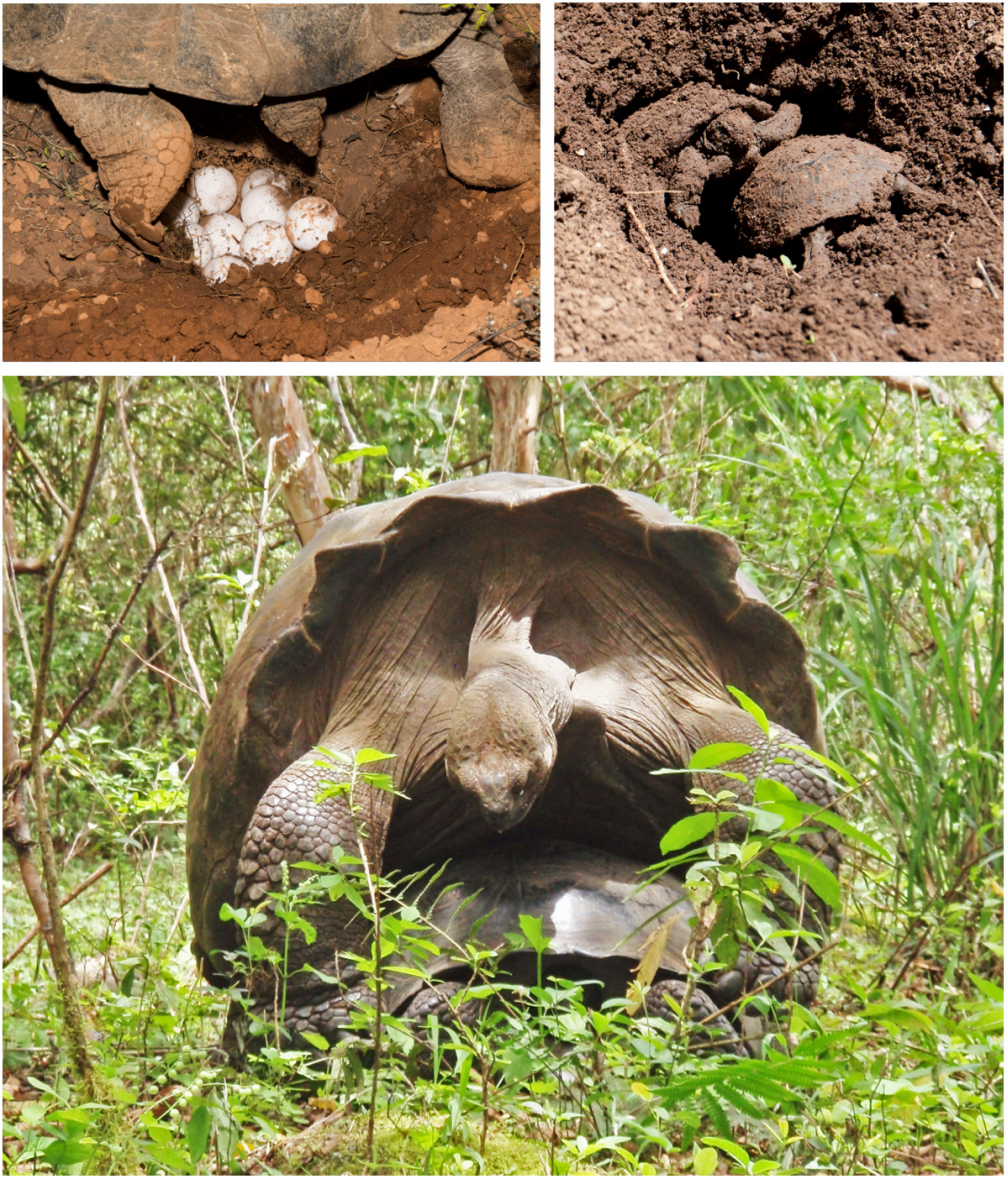
When these Galapagos tortoise eggs were laid, the sexes of the embryos were undetermined. At a crucial moment during incubation, the sex of each embryo became set. Embryos experiencing temperatures above the “pivotal temperature” became females while embryos experiencing lower temperatures became males. Adult female tortoises that lay their eggs at cooler, usually higher elevations on the volcanic slopes of islands in the Galapagos Archipelago are more likely to produce male offspring compared to females that nest at lower, warmer elevations. Climate change and the nesting decisions made by females will strongly influence the future of giant Galapagos tortoise sex ratios and population demographics.

Impacts of climate change are manifested across the tree of life, but can be particularly potent for ectotherms, which make up the majority of animal biodiversity (Wilson, 1992). For these taxa, environmental temperature determines metabolic rate, which governs patterns of growth, reproduction, locomotion efficiency, and immune responses (Schmidt‐Nielsen, 1997). For many ectothermic species, climate change is already profoundly impacting population dynamics, ecology, behavior, and morphology (Deutsch et al., 2008, 2015; Gunderson & Leal, 2016; Kearney et al., 2009; Kingsolver et al., 2013; McCauley et al., 2018). For some ectothermic taxa, including many reptiles and teleost fish, rapidly changing environmental temperatures may influence sex determination during development, and therefore sex ratios of populations (Janzen, 1994; Mitchell & Janzen, 2010; The Tree of Sex, 2014). Temperature‐dependent sex determination (TSD) occurs in some lizards, all crocodilians, and most turtle species (Janzen & Paukstis, 1991). Three patterns are recognized based on one or two “pivotal temperatures,” or those temperatures that give approximately equal numbers of males and females, (Bull, 1980): (A), in which high and (B) low incubation temperatures result in females; and (C), with two pivotal temperatures, in which low and high temperatures produce females, and intermediate temperatures produce males. Temperature‐dependent sex determination may have evolved in lineages in which temperature during development influences male and female fitness differently (Charnov & Bull, 1977). The three reptile lineages (Lepidosauria, chelonian, crocodiles) which display TSD differentiated from a common ancestor over 200 million years ago. Since this time, mean global temperatures have fluctuated considerably (10–20°C) and over a few decades during the Holocene yet many lineages with TSD survived (Mitchell & Janzen, 2010). Rapid temperature fluctuation over geological time was limited mostly to the northern hemisphere, which may have allowed some taxa to persist (Mitchell & Janzen, 2010). Others survived changing thermal conditions through adaptation and/or phenotypic plasticity (Refsnider & Janzen, 2016). Current climate change is comparable in rate (Dt = 0.2–0.6°C per decade) and magnitude (Dt by 2100 = 1.3–5.7°C above current mean) (Wuebbles et al., 2017) but is global in scale. What is not comparable is the conservation status of species and populations that display TSD during the current rapid phase of global warming (Figure 1).

The Chelonia (turtles and tortoises) is the most imperiled vertebrate order, with more than half (61%) of the 356 known species threatened with extinction due to over‐harvesting (e.g., illegal trade), infectious diseases, habitat destruction, and climate change (Lovich et al., 2018; Rhodin et al., 2021). Of the 79 chelonian species tested, over 80% display TSD (Ewert et al., 2004). Species with TSD are composed of small, endangered populations that often face multiple threats, with low genetic and phenotypic diversity, may lack the resilience to withstand the impact of rapid temperature change on sex ratios, effective population size, or emergence time/food availability mismatch (Mitchell & Janzen, 2010). A growing body of empirical and modeling evidence demonstrates that nest temperatures of marine and freshwater turtles are increasing under climate change and skewing sex ratios (Fuentes & Porter, 2013; Hawkes et al., 2009; Jensen et al., 2018; Patrício et al., 2021; Refsnider et al., 2013; Santidrián Tomillo et al., 2015). It is likely that terrestrial Chelonians are, or will be, similarly impacted as has been shown in the red‐footed tortoise (Hernández‐Montoya et al., 2017).

Among chelonians, Galapagos tortoises (*Chelonoidis* spp.) are conservation icons and emblematic of terrestrial tortoises worldwide. Of the 14 known Galapagos tortoise species, at least 12 remain extant, of which six are critically endangered, three are endangered, and three are vulnerable (IUCN, 2022). Although uncertain, overall predictions for the Galapagos trend to a warmer and wetter climate with both El Niño and La Niña (ENSO) events more frequent and intense (Restrepo et al., 2012; Trueman & d'Ozouville, 2010). Changing temperature and precipitation will have long‐term impacts on Galapagos flora and fauna (Charney et al., 2021), with recent evidence of rapid anthropogenic environmental changes in Galapagos, including climate, triggering intense selection pressures for adaptive phenotypes and rapid evolution (Deem et al., 2010; Grant et al., 2017; Grant & Grant, 2003). More generally, the potential for rapid evolution and/or phenotypic plasticity to keep pace with environmental change is a concern globally and across the tree of life as the Anthropocene proceeds (Carroll et al., 2014; Johnson & Munshi‐South, 2017). This challenge is perhaps greatest for long‐lived, long‐generation time endemic species on islands comprised of small, isolated populations that have experienced one or more genetic bottlenecks (Graham et al., 2017). All these traits are exemplified by giant Galapagos tortoises (Jensen et al., 2021; Poulakakis et al., 2012).

Giant tortoises occur on seven of the Galapagos Islands (Caccone et al., 2002) (eight with a recently introduced population to Santa Fe Island) (Tapia et al., 2022). The topography of the Galapagos Islands varies from relatively flat, older islands to young active volcanoes reaching maximum elevation of 1900 m above sea level (ASL) (Tye et al., 2002). Tortoises occur across the entire elevation gradient. Female nest site selection varies by island and within populations and can occur from close to sea level to at least ca. 1000 m ASL (SB pers. obs.). The combination of large seasonal, interannual, and elevational variation in insolation, air temperature, and vegetation cover are likely to have a strong influence on soil temperature and therefore potentially on hatchling sex ratios; however, this has never been documented. Understanding patterns of sex determination among recruits into populations, and the drivers of these patterns is a high research priority (Kubisch & Ibargüengoytía, 2021).

**FIGURE 2:**
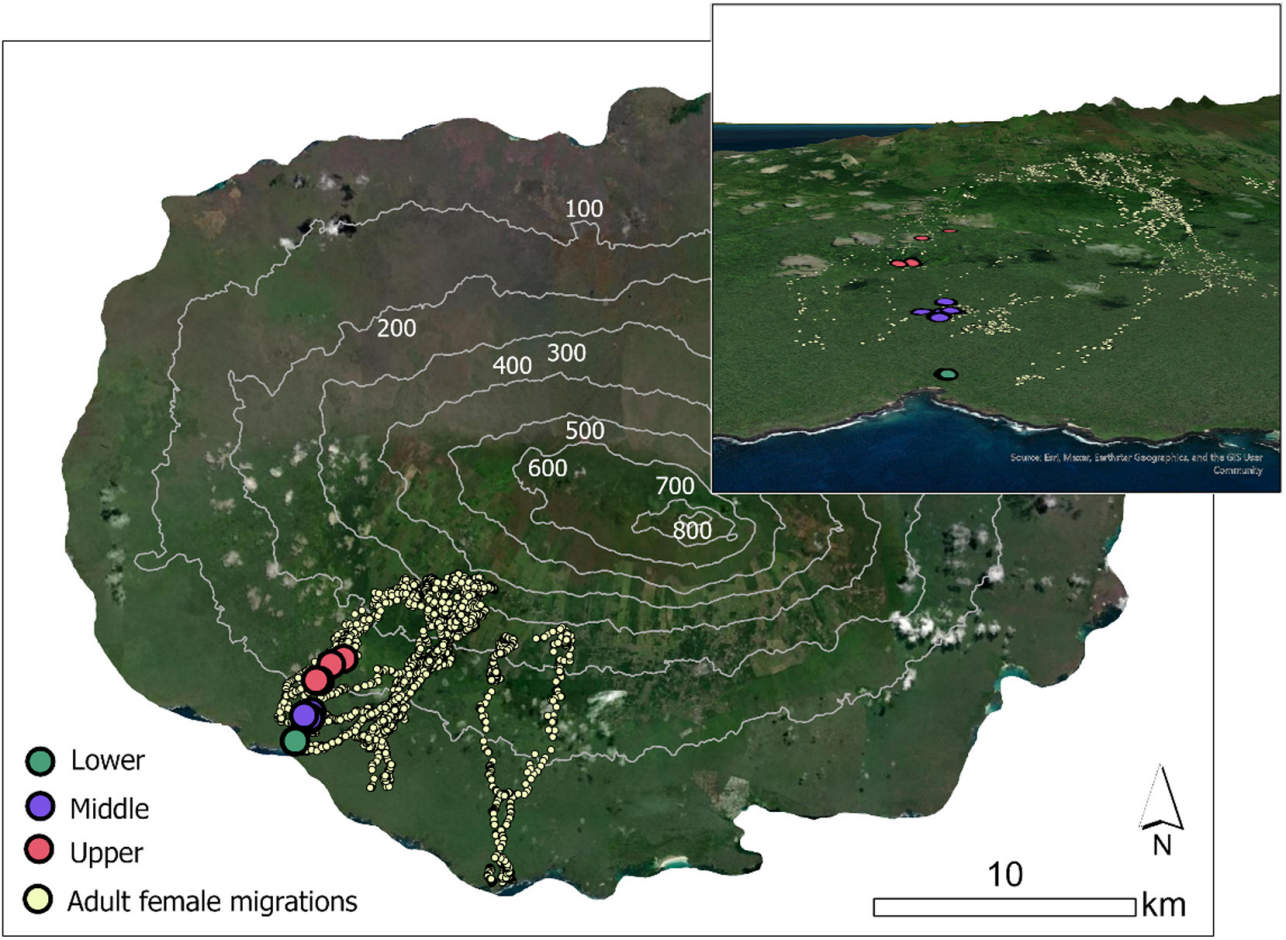
Locations of three Galapagos tortoise nesting zones in the El Chato region of Santa Cruz Island, Galapagos. Seasonal migration data of adult female tortoises are from Yackulic et al. (2017). Map insert exaggerates elevation three‐fold.

From studies conducted in captivity, Galapagos tortoises are known to display the A pattern of TSD with warmer temperatures producing females and cooler temperatures producing males (Marquez et al., 1990; Sancho et al., 2017). In the Tortoise Reserve on Santa Cruz Island at least six tortoise nesting areas are known to rangers from the Galapagos National Park Service. Within one of these areas, El Chato, three distinct nesting zones occur along an elevational gradient between 10 and 140 m ASL (Figure 2), which are included in a long‐term study on Galapagos tortoise movement ecology (Blake et al., 2013; Blake, Yackulic, et al., 2020). Application of a thermal model developed by Blake, Parlin, et al. (2020) indicates a ca. 1°C decline in mean annual ambient air temperature from lower to upper nesting zone and more persistent cloud cover decreases direct solar radiation as elevation increases (Trueman & d'Ozouville, 2010). While air and soil temperatures are correlated (Blake, Parlin, et al., 2020), variation in nest temperatures over the elevation gradient are unknown, which motivated this study.

We hypothesized that the temperature gradient over three nesting zones would result in a higher frequency of female hatchlings produced in warmer, lower elevation nests, and more male hatchlings produced in the cooler, upper elevation nests. To test our hypothesis, we measured nest temperatures and determined the sex of juvenile tortoises residing in the three nesting zones along the elevational gradient. Here we discuss the implications of our results for Galapagos tortoises within the context of projected climate change in the Galapagos archipelago.

## 2 METHODS

### 2.1 Study site

The Galapagos archipelago lies ca. 1000 km west of continental Ecuador in the Eastern Tropical Pacific Ocean. Santa Cruz Island, in the center of the archipelago, is inhabited by two species of Galapagos tortoises, the Western Santa Cruz tortoise (*Chelonoidis porteri*) which occurs on the island's southwestern flank, including within the “Tortoise reserve,” and the Eastern Santa Cruz tortoise (*Chelonoidis donfaustoi*) (Poulakakis et al., 2012) to the southeast of the island. This study was conducted in the Tortoise Reserve (El Chato) (Figure 2) and thus only included *C. porteri*. The site contains three distinct nesting zones along the elevational gradient: Lower (14 m mean elevation), Middle (57 m mean elevation), and Upper (107 m mean elevation), which are separated by regions of extensive lava rock. Our ongoing telemetry study strongly indicates that juvenile tortoises up to 8 years old do not move between these nesting zones (Blake et al., In second review) thus the sex ratios of samples of juveniles found in each zone reflect the hatchling sex ratio of that zone.

Juvenile tortoises were located within each of the three nesting zones by either radio telemetry (*n* = 15) of tagged animals as part of an ongoing study (Blake, Yackulic, et al., 2020) or opportunistically (*n* = 25) via searches on foot conducted between July 4 and 13, 2017. For each juvenile tortoise sampled, we obtained capture location using GPS, placed it in a clean cotton bag and carried it to a field laboratory where coelioscopy was carried out. Coelioscopic sexing was performed using a 2.7‐mm‐diameter 30° rigid endoscope within a protective sheath (Karl Storz Endoscopy‐America) attached to an Endogo system (Envisionier Medical Technologies) in anesthetized tortoises, as described by Emmel et al. (2021).

We located 54 freshly completed nests across the three zones (12 in the lower zone, 23 in the middle zone, and 19 in the upper zone) over a five‐year period (2013–2017), during which we recorded internal nest temperatures. Each fresh nest (based on first observation of nest presence at our field site and visual assessment of soil consistency) was opened, carefully removing the nest cap intact with a machete and digging down into the chamber by hand. The eggs were marked, removed, and placed on the soil by the nest without changing the orientation of the egg. We estimated the center of the egg chamber by eye and measured its depth below the soil surface, placing an iButton thermochron (temperature loggers, DS1922L, Maxim Integrated Products) on top of the eggs at that depth. iButtons were programmed to collect temperature data to the nearest 0.5°C every 4 h. We replaced all eggs in their original location and orientation using soil from the nest to stabilize them where appropriate. Finally, we re‐packed soil over the uppermost eggs, replaced the nest cap, and sealed it with the remaining soil.

### 2.2 Analysis

Sex ratio (estimated proportion of males) along the elevation gradient was modeled using logistic regression with a logit link, and nesting zone mean elevation and juvenile weight (grams) as independent variables. Juvenile weight was included to rule out differential survival by sex as an influencing factor on the observed sex ratios. If males and females had different survival rates, our sample would be biased to the sex with highest survival, and potentially confound the effect of temperature. Data limitations (too few males at low elevations and too few females at higher elevations) precluded a test of the effect of an interaction between elevation and juvenile weight on sex ratio. The hypothesis that incubation temperature decreased with increasing elevation was tested using a Generalized Linear Model (GLMM) with mean nest temperature per 1% time increment through incubation as the dependent variable, nesting zone, and year (hatchling cohort) as fixed effects, and nest and percent incubation time as random effects. To calculate the 1% increments of incubation time we took the estimated total incubation time and divided this time into 100 equal units. We did not know exact dates of nest laying or egg hatching. We used the percentage time rather than number of days because the number of days of incubation varied for each nest as a function of temperature. Nest temperatures were quantified throughout incubation, but because sex is determined during the middle trimester of incubation among chelonians (Bull, 1980), only temperature values between 33% and 66% of the incubation period were included in analyses. Statistical analyses were completed in Genstat 18 (VSN International Ltd.). All animal handling and procedures followed guidelines from the Galapagos National Park Directorate under Research Permit PC‐36‐17 and Institutional Animal Care and Use Committee (IACUC) protocol 2017–01 of the Saint Louis Zoo for coelioscopy.

## 3 RESULTS

### 3.1 Sex determination

**FIGURE 3:**
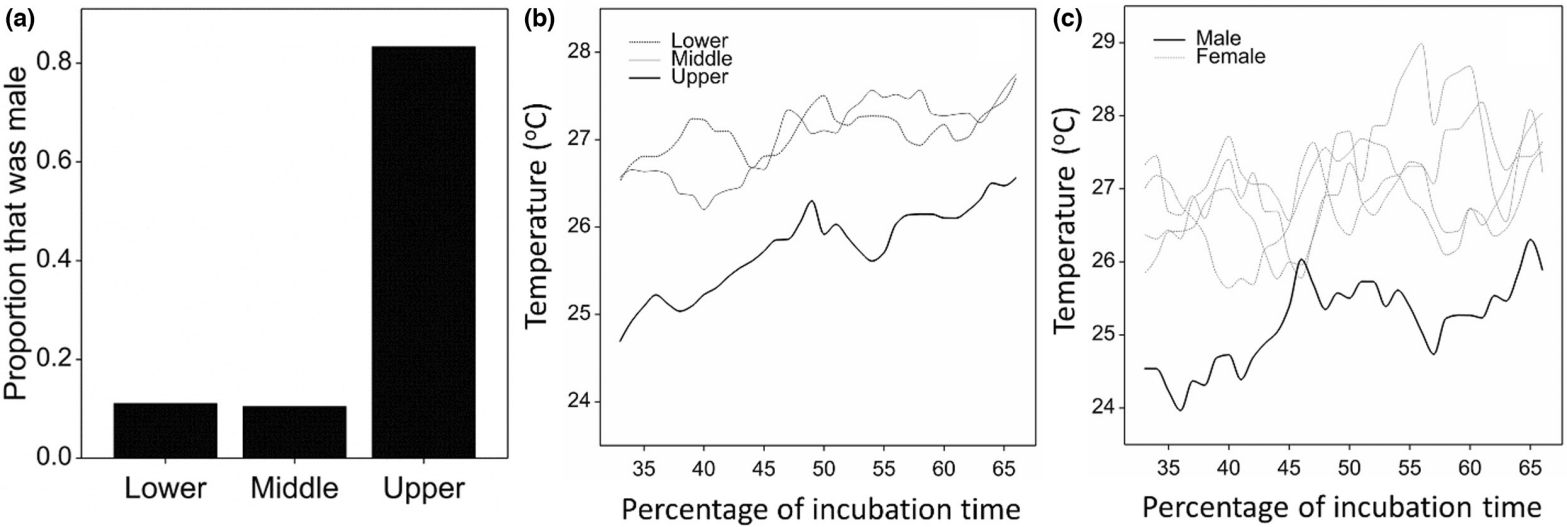
(a) The majority (80%) of juvenile Western Santa Cruz tortoises in the upper nesting zone were males, while in the middle and lower nesting zones, males made up only 11.1% and 9.5% of juveniles. (b) Temperatures of nests varied considerably through the middle of incubation but were considerably cooler in the upper nesting zone, where males dominated the juvenile tortoise sex ratios. (c) Nest temperatures of juveniles from known nests were higher for four nests that produced females compared to a nest that produced a male. In all graph line elements, splines with 50 degrees of freedom are used for illustrative purposes.

Juveniles were collected at elevations between 10 and 112 m ASL, and temperature data were collected from nests between 6 and 149 m ASL. All juveniles appeared in good physical condition on visual examinations. We successfully identified the sex of 40 animals from the three nesting zones (9, 21, and 10 from the lower, middle, and upper zones, respectively). Body weights ranged from 77 to 1358 g, with a mean of 529 g. Of 9 and 21 juveniles collected and sexed in the lower and middle nesting zones, respectively, only one (11.1%) and two (9.5%) were male, while from the upper nesting zone, eight out of ten (80%) juveniles were male (Figure 3a). In support of our hypothesis, a logistic regression indicated a significant positive relationship between increasing nest elevation and the probability of being male (Deviance Ratio _(2,37)_ = 8.43, *p* < .001). Juvenile weight had no significant effect on sex ratios, suggesting that incubation temperature was the primary determinant of sex ratios. Parameter estimates were as follows: Elevation = 0.04 (SE = 0.0167), *p* = .017; Juvenile weight = −0.00256 (SE = 0.00184), *p* = .163.

### 3.2 Nest temperatures

During the middle trimester of incubation, mean nest temperature decreased significantly with increasing elevation supporting our hypothesis (*F* = 37.23, ddf = 47.0, *p* < .001) (Figure 3b). The mean difference in temperature was small between the lower and middle nesting zones (mean = 0.02°C), but considerable between the middle and upper nesting zones (mean = 1.57°C). Year did not have a significant effect on mean nest temperature (*F* = 2.04, ddf = 47, *p* < .103), with low interannual differences; 0.39°C between 2014 and 2015, and 0.79°C between 2014 and 2016.

Of all juveniles evaluated by coelioscopy, five (one male and four females) were incubated in nests with associated nest temperature data. For these five nests, incubation temperatures during the third trimester were variable, however, the nest from which the male emerged was 1.7°C (SD = 0.69°C) cooler than the nests that produced females (Figure 3c).

## 4 DISCUSSION

Consistent with our hypothesis, we demonstrated that (1) nest temperatures of free‐living *C. porteri* were negatively correlated with elevation, and (2) the frequency of male juvenile tortoises in the population was positively correlated with increasing elevation. It is noteworthy that the total elevational change across all nesting zones was little over 100 m, and the switch from ca. 10% males to >80% males occurred between 57 m and 104 m mean nest elevation.

We recorded considerable fluctuation in nest temperature both diurnally and over longer periods, though generally mean nest temperature increased throughout the incubation period. The impacts of these variable thermal regimes on sex determination (and indeed development and survival more generally) are not known for this species. The highly variable thermal regimes of nests in the field and our very small sample sizes preclude any attempt to define a pivotal temperature (Ewert et al., 1994, 2004; Janzen & Paukstis, 1991; Warner & Shine, 2011). Sancho et al. (2017) estimated a pivotal temperature of 28.3°C for captive Española Island tortoises (*Chelonoidis hoodensis*), based on empirical data in which a consistent incubation temperature of 25.5°C produced 91% male hatchlings, while 33% males and 67% females resulted from an incubation temperature of 29.5°C. The Sancho et al. (2017) model predicted a large “transition range” from 25.2°C to 31.4°C over which both males and females would be produced. A large transition range would provide considerable buffering against potentially catastrophic demographic impacts of climate change (Sancho et al., 2017). However, the relatively small range of mean incubation temperature and dramatic change in sex ratio demonstrated in our study suggests a much narrower transition temperature range.

The difference between the nest temperatures we recorded, and the incubation temperatures used at the Tortoise Breeding Centers on Galapagos (28°C for males, 29.5°C for females) is noteworthy. In our study, mean nest temperatures taken in the middle of the nesting chambers rarely exceeded 28°C, and never surpassed 29.5°C during the middle trimester of incubation (Figure 2b,c). There are no published data on the outcome of these incubation temperatures (i.e., below 28°C and above 29.5°C) from the Captive Breeding Centers: however, our data would suggest that tortoises incubated in captivity may have sex ratios heavily skewed toward females.

We attributed the change in sex ratios across the nesting zones to differences in incubation temperatures. We can rule out differential mortality of males and females as the cause of sex ratio differences observed because mean body size by sex was not significantly different across nesting zones. However, our data do not allow us to rule out sex‐related egg mortality differences by zone, though this seems unlikely.

Our study was motivated not only by obtaining the first indication of how nest location may influence nest temperature and sex ratios of offspring but also by the question of how a changing climate may impact Galapagos tortoise sex ratios, and the potential consequences for tortoise demography and conservation efforts. Our study was limited to three nesting zones in a small part of the range of one species, so we are unable to generalize to the population level, species, or beyond across the archipelago. We do not know current sex ratios of populations or species, nor do we know the full distribution of nest sites or their thermal regimes during incubation. However, we can predict that the projected increase in ambient temperature of Galapagos of between 1 and 4°C (Charney et al., 2021) will highly skew the sex ratio toward females. In the short term, more females will likely increase population growth rates (Blechschmidt et al., 2020; Butler, 2019; Deutsch et al., 2008), since the abundance and survival of reproductively active females largely determines population growth rate in long‐lived species such as Galapagos tortoises (Gibbs & Goldspiel, 2020). However, a dramatic reduction in male recruitment would further erode genetic diversity and reduce effective population size (Reed & Frankham, 2003) of these already genetically compromised species (Milinkovitch et al., 2004, 2013; Russello et al., 2005). An alternative outcome may be related to predictions of increased precipitation that would stimulate vegetation cover and reduce direct solar radiation onto the soil. Potentially this could counter ambient warming and reduce nest temperatures favoring male hatchlings. However, the projected increases in rainfall is also likely to have a serious negative impact on egg and hatchling mortality, over‐riding the effects of sex ratio change in many parts of Galapagos (Charney, 2021).

Galapagos tortoises on some islands nest anywhere between 100 and 1000 m elevation, but we can make no general predictions on sex ratios with elevation from our data because interactions between elevation, aspect, sea surface temperature, and dry season cloud cover generate heterogeneous temperature profiles (Blake, Parlin, et al., 2020). There is a huge geographic range over which Galapagos tortoises could potentially nest to optimize fitness (which includes offspring sex ratios) in a changing climate. However, we do not know to what extent Galapagos tortoises are philopatric to nest sites or whether females can make decisions on where to nest based on current climate and other factors. Studies of chelonians elsewhere suggest that strong philopatry is common (Freedberg et al., 2005; Lee et al., 2007; Stiebens et al., 2013; Walde et al., 2007) which may limit a plastic response to nest site location by females as a response to rapid climate change. If nest site choice does exhibit plasticity, Galapagos tortoise species that live on islands with a large elevation range may be able to shift their nesting sites to higher, cooler, elevations to maintain consistent sex ratios under climate change. In fact, behavioral plasticity and rapid evolutionary response may have potential to mitigate catastrophic climate impacts for some species (Gunderson & Leal, 2016; Kingsolver et al., 2013). Furthermore, plasticity among female chelonians in nest depth has been suggested as a possible mechanism to maintain stable sex ratios in response to climate variability, though conclusions are equivocal (Marco et al., 2018; Ozdemir et al., 2011; Refsnider et al., 2013).

## 5 CONCLUSION

This is the first study to quantify the potential impact of temperature on sex ratios of Galapagos tortoises in the wild. We demonstrated that a small (ca. 50 m) increase in elevation induced a significant drop in nest temperatures, and we documented a flip in sex ratios of juveniles from ca. 10% males at low elevations to 80% males at the upper elevation. Over the next 30–50 years, mean temperatures on Galapagos are predicted to become hotter than at any time over the last 120,000 years (Charney et al., 2021). This will likely increase the F:M ratio in many nesting zones, but the overall consequences for populations and species of Galapagos tortoises cannot be determined because of several unknown factors including: 1. the distribution of nest sites, 2. the thermal regimes of nests over time and space, 3. pivotal and transitional temperatures, and 4. the range of behavioral plasticity among nesting females. Nonetheless, our study highlights the need to include the potential impact of TSD on Galapagos tortoise demographics in conservation planning and management of these iconic species.

## AUTHOR CONTRIBUTIONS

**Sharon L. Deem:** Conceptualization (equal); data curation (equal); formal analysis (equal); funding acquisition (equal); investigation (equal); methodology (equal); project administration (equal); resources (equal); supervision (equal); visualization (equal); writing – original draft (equal); writing – review and editing (equal). **Sam Rivera:** Formal analysis (equal); investigation (equal); methodology (equal); resources (equal); writing – review and editing (equal). **Ainoa Nieto‐Claudin:** Investigation (equal); methodology (equal); project administration (equal); writing – review and editing (equal). **Evan Emmel:** Investigation (equal); methodology (equal); writing – review and editing (equal). **Freddy Cabrera:** Investigation (equal); methodology (equal). **Stephen Blake:** Conceptualization (equal); data curation (equal); formal analysis (equal); funding acquisition (equal); investigation (equal); methodology (equal); project administration (equal); resources (equal); visualization (equal); writing – original draft (equal); writing – review and editing (equal).

## FUNDING INFORMATION

This work was supported through Saint Louis Zoo Institute for Conservation Medicine, the Galapagos Conservation Trust, Houston Zoo, The Swiss Friends of Galapagos, the National Geographic Society Committee for Research and Exploration, and the National Science Foundation (DEB 1258062). VSN International (Hemel Hempstead, UK) provided free statistical software.

## CONFLICT OF INTEREST STATEMENT

We declare that we have no competing interests.

## Data Availability

Upon publication, all datasets used in this paper will be curated in Dryad under orcid.org code 0000‐0002‐2220‐1589.
